# Genes Bound by ΔFosB in Different Conditions With Recurrent Seizures Regulate Similar Neuronal Functions

**DOI:** 10.3389/fnins.2020.00472

**Published:** 2020-05-28

**Authors:** Gabriel S. Stephens, Chia-Hsuan Fu, Corey P. St. Romain, Yi Zheng, Justin J. Botterill, Helen E. Scharfman, Yin Liu, Jeannie Chin

**Affiliations:** ^1^Memory and Brain Research Center, Department of Neuroscience, Baylor College of Medicine, Houston, TX, United States; ^2^Center for Dementia Research, Nathan Kline Institute for Psychiatric Research, Orangeburg, NY, United States; ^3^Departments of Child & Adolescent Psychiatry, Neuroscience & Physiology, and Psychiatry, New York University Neuroscience Institute, New York University Langone Health, New York, NY, United States; ^4^Department of Neurobiology and Anatomy, University of Texas Medical School at Houston, Houston, TX, United States

**Keywords:** epilepsy, Alzheimer’s disease, seizure, deltaFosB, epigenetic, pilocarpine, ChIP-seq, hippocampus

## Abstract

Seizure incidence is increased in Alzheimer’s disease (AD) patients and mouse models, and treatment with the antiseizure drug levetiracetam improves cognition. We reported that one mechanism by which seizures can exert persistent effects on cognition is through accumulation of ΔFosB, a transcription factor with a long half-life. Even the infrequent seizures that spontaneously occur in transgenic mice expressing human amyloid precursor protein (APP) lead to persistent increases in ΔFosB in the hippocampus, similar to what we observed in patients with AD or temporal lobe epilepsy. ΔFosB epigenetically regulates expression of target genes, however, whether ΔFosB targets the same genes when induced by seizures in different neurological conditions is not clear. We performed ChIP-sequencing to assess the repertoire of ΔFosB target genes in APP mice and in pilocarpine-treated wildtype mice (Pilo mice), a pharmacological model of epilepsy. These mouse models allowed us to compare AD, in which seizures occur in the context of high levels of amyloid beta, and epilepsy, in which recurrent seizures occur without AD-specific pathophysiology. Network profiling of genes bound by ΔFosB in APP mice, Pilo mice, and respective control mice revealed that functional domains modulated by ΔFosB in the hippocampus are expanded and diversified in APP and Pilo mice (vs. respective controls). Domains of interest in both disease contexts involved neuronal excitability and neurotransmission, neurogenesis, chromatin remodeling, and cellular stress and neuroinflammation. To assess the gene targets bound by ΔFosB regardless of seizure etiology, we focused on 442 genes with significant ΔFosB binding in both APP and Pilo mice (vs. respective controls). Functional analyses identified pathways that regulate membrane potential, glutamatergic signaling, calcium homeostasis, complement activation, neuron-glia population maintenance, and chromatin dynamics. RNA-sequencing and qPCR measurements in independent mice detected altered expression of several ΔFosB targets shared in APP and Pilo mice. Our findings indicate that seizure-induced ΔFosB can bind genes in patterns that depend on seizure etiology, but can bind other genes regardless of seizure etiology. Understanding the factors that underlie these differences, such as chromatin accessibility and/or abundance of co-factors, could reveal novel insights into the control of gene expression in disorders with recurrent seizures.

## Introduction

Seizures and epileptiform activity accompany several acute and chronic conditions, including temporal lobe epilepsy (TLE), and other epilepsies, Alzheimer’s disease (AD), amnestic mild cognitive impairment (aMCI), autism spectrum disorders, autoimmune disorders, stroke, traumatic brain injury, and others (Bell et al., 2011; Vossel et al., 2013, 2016, 2017; Mahler et al., 2015; Bernard, 2016; Buckley and Holmes, 2016; Pitkanen et al., 2016; Bien and Holtkamp, 2017; Lam et al., 2017). Seizures can regulate diverse cellular pathways that control neuronal excitability and other domains of function (Kleen et al., 2012; Noebels J., 2015; Noebels J. L., 2015; Bernard, 2016). It is therefore crucial to identify pathways that may control seizures and/or downstream impacts of seizures on neurological function.

Even infrequent seizures can lead to persistent deficits in neurological function by altering network dynamics and gene regulatory pathways (Kleen et al., 2012; Chin and Scharfman, 2013; Holmes, 2015; Noebels J. L., 2015; Bernard, 2016; Buckley and Holmes, 2016; Vossel et al., 2017). Seizures can induce long-lasting epigenetic changes that sustain persistent changes in gene expression even during seizure-free periods (Roopra et al., 2012; Bernard, 2016; Corbett et al., 2017; You et al., 2017; Kobow and Blumcke, 2018; You et al., 2018). Epigenetic regulation by seizures can occur through mechanisms that include DNA methylation and histone modification. DNA methylation involves covalent bonding of methyl groups to cytosine residues and primarily occurs at genomic CpG sites (cytosine followed by guanine), typically in ways that promote formation of closed areas of repressed heterochromatin and that reduce open areas of active euchromatin (Allis and Jenuwein, 2016). Histone modifications that regulate chromatin architecture and gene expression include acetylation and methylation of histone tails. Histone acetyltransferases (HATs) and histone deacetylases (HDACs) add and remove acetyl groups, respectively, and methyltransferases (HMTs) and demethylases (HDMs) add and remove methyl groups, respectively. Seizures can also change chromatin architecture through less-studied epigenetic mechanisms that involve histone ubiquitination, regulation by non-coding RNAs, or ATP-dependent physical rearrangement of chromatin loops by remodeling factors (Kobow and Blümcke, 2014). Seizure-induced transcription factor activity can control these epigenetic mechanisms (e.g., by recruiting HDACs or other factors to regions of DNA) and thereby impact cellular function in conditions with seizures. For example, the transcription factor repressor element 1-silencing transcription factor (REST) is induced by seizure activity and epigenetically regulates histone modification and expression of target genes in ways that promote further epileptogenesis (Roopra et al., 2012; McClelland et al., 2014).

We recently identified a critical role for another activity-induced transcription factor, ΔFosB, in maintaining long-lasting epigenetic regulation of gene expression in conditions with spontaneous recurrent seizures, including both temporal lobe epilepsy and AD (Corbett et al., 2017; You et al., 2017, 2018). ΔFosB is a leucine zipper that can bind to target genes in complex with other AP-1 factors and can control diverse pathways by regulating target gene expression (Nestler and Luscher, 2019). A truncated splice variant of the *FosB* gene, ΔFosB has a long half-life (∼8 days *in vivo*) due to the lack of two C-terminal degron domains that target it for proteasomal degradation, and with increased phosphorylation that enhances its stability; these features allow ΔFosB to accumulate with chronic neuronal activity (Carle et al., 2007; Ulery-Reynolds et al., 2009; Nestler and Luscher, 2019). ΔFosB can recruit histone-modifying proteins to target genes, a process that has been well-characterized in the nucleus accumbens (NAc), in which ΔFosB accumulates after chronic exposure to drugs of abuse and regulates gene expression (Renthal et al., 2008; Robison and Nestler, 2011; Nestler and Luscher, 2019). Our work demonstrated that seizures induce ΔFosB accumulation in the hippocampus of two mouse models with spontaneous recurrent seizures: pilocarpine-treated mice (Pilo mice) as well as transgenic mice that express human amyloid precursor protein (APP) carrying mutations linked to AD (line J20) (Corbett et al., 2017; You et al., 2017; Fu et al., 2019). Importantly, we showed that ΔFosB also accumulates in the hippocampus of patients with AD and in patients with temporal lobe epilepsy (TLE) (You et al., 2017). In APP and Pilo mice, seizure-induced ΔFosB bound to target genes encoding c-Fos and calbindin, led to deacetylation and hypermethylation of histone H4, and reduced target gene expression (Corbett et al., 2017; You et al., 2017). These epigenetic alterations contributed to persistent cognitive deficits, even when seizure activity was infrequent.

Because seizure-induced ΔFosB can sustain persistent cognitive deficits through regulation of its target genes, it is crucial to identify all gene targets by which ΔFosB can alter neuronal function. We previously used ΔFosB ChIP-sequencing analyses to identify and characterize ΔFosB target genes in the hippocampus of young (pre-plaque) APP mice and non-transgenic (NTG) littermates. We reported that the repertoire of ΔFosB target genes largely differed between APP and NTG mice. ΔFosB target genes in APP mice were more numerous and were involved in epilepsy-relevant functions including excitability and neurogenesis, whereas ΔFosB target genes in NTG mice were less numerous and were involved in basal functions like maintenance of cell polarity (You et al., 2018). That ΔFosB binds to different targets in APP vs. NTG mice suggests that ΔFosB may regulate different sets of target genes depending on context. However, we wondered whether seizure-induced ΔFosB would target the same set of genes regardless of seizure etiology.

To determine how ΔFosB target binding compares between conditions with different seizure etiologies, we performed ChIP-seq analyses to identify target genes bound by ΔFosB in young APP mice and in Pilo mice with chronic recurrent seizures. Young APP mice at 4 months of age exhibit spontaneous recurrent seizures that begin around 2–2.5 months of age, and cognitive deficits, but do not yet exhibit overt amyloid plaque pathology or neurodegeneration; these mice were compared with Pilo mice at 5 weeks after induction of status epilepticus, at which point they have spontaneous recurrent seizures and are considered to be in the chronic phase (Mucke et al., 2000; Palop et al., 2007; Scharfman, 2012; Levesque et al., 2016; Corbett et al., 2017; You et al., 2017; Fu et al., 2019). This approach allowed us to identify ΔFosB target genes that are bound in the chronic phase of epilepsy with seizures of different etiologies. We found that ΔFosB target genes were more numerous in either APP or Pilo mice vs. respective control mice. Moreover, in APP mice or in Pilo mice, ΔFosB target genes were largely represented by 4 categories: excitability and neurotransmission, neurogenesis, chromatin remodeling, and cellular stress and immunity. When we examined the list of target genes that overlapped in APP and Pilo mice, we found that the same 4 categories were also represented. RNA-sequencing and RT-qPCR analyses demonstrated that a number of ΔFosB target genes showed altered expression in APP mice and in Pilo mice relative to respective controls. Together, our results indicate that seizure-induced ΔFosB can bind target genes in seizure etiology-dependent and -independent patterns. Characterization of genes bound by ΔFosB regardless of seizure etiology may be valuable for identifying common pathways that might regulate neuronal function across multiple disease contexts.

## Materials and Methods

### Transgenic APP Mouse Model of Alzheimer’s Disease (AD)

We used transgenic mice expressing human amyloid precursor protein (APP) carrying the Swedish (K670N, M671L) and Indiana (V717F) mutations (Line J20; hAPP770 numbering) (Mucke et al., 2000) driven by the platelet-derived growth factor (PDGF) β chain promoter. The line was backcrossed over 10 generations onto a C57BL/6 background, with heterozygosity maintained via breeding with wild-type C57BL/6 mice from The Jackson Laboratory. Age- and sex-matched wild-type non-transgenic (NTG) mice were used as littermate controls. Mice were housed with *ad libitum* access to a diet of LabDiet 5V5R chow, in cages with corn cob bedding and EnviroPak nesting material, on a 12:12 light/dark cycle. Mice were maintained group housed 4-5/cage until appropriate ages for experimental studies, at which point they were singly housed for 2 days prior to sacrifice and brain harvesting. 4-month old APP and NTG mice were used in this study for ChIP-sequencing because at this age APP mice produce high levels of Aβ but do not yet exhibit overt amyloid plaque pathology nor neurodegeneration, but do exhibit both non-convulsive and convulsive seizures (beginning around 2–2.5 months of age) as well as cognitive deficits (Palop et al., 2007; Sanchez et al., 2012; Chin and Scharfman, 2013; Corbett et al., 2017; You et al., 2017; Fu et al., 2019). Therefore, by 4 months of age, APP mice have typically experienced seizures for 6–8 weeks, which is in line with the chronic phase of epilepsy in other models. To extract tissue, mice were anesthetized with isoflurane and then transcardially perfused with ice-cold saline before brains were removed and hemisected. The right side hemibrain was post-fixed for 48 h in 4% paraformaldehyde in phosphate-buffered saline at 4°C prior to storage in phosphate-buffered saline at 4°C, and the left side hemibrain was flash-frozen in dry ice and stored at -80°C for biochemical experiments. All experiments were carried out in accordance with recommendations in the Guide for the Care and Use of Laboratory Animals of the National Institutes of Health and under protocol AN-6943 approved by the Baylor College of Medicine Institutional Animal Care and Use Committee (IACUC).

### Pilocarpine-Induced Model of Temporal Lobe Epilepsy (TLE) and Recurrent Seizure Activity

Male and female 3–4-month old wild-type C57BL/6 mice from Charles River Laboratories (Wilmington, MA, United States) were group housed, with *ad libitum* access to a diet of Purina 5008 chow, in cages with corn cob bedding, on a 12:12 light/dark cycle. For pilocarpine-induced recurrent seizure activity, mice were initially administered scopolamine methylnitrate and terbutaline hemisulfate (each 2 mg/kg subcutaneous (s.c.); Sigma-Aldrich) to, respectively, inhibit peripheral effects of pilocarpine and dilate respiratory tracts. In this pretreatment, mice were also injected with ethosuximide, a T-type Ca^2+^ channel inhibitor (150 mg/kg s.c.; Sigma-Aldrich) that we have found to be effective at reducing mortality after seizure induction in C57BL/6 mice (Iyengar et al., 2015). Thirty minutes after pretreatment, mice were injected with either saline (Sal mice) or pilocarpine hydrochloride (Pilo mice, 240–250 mg/kg s.c.; Sigma-Aldrich), and then placed in a heated cage maintained at 31°C (Cat# S12781, Animal Intensive Care Unit, Thermocare, Paso Robles, CA). Acute seizures were behaviorally monitored using a modified Racine’s scale: stage 1, mouth and facial movements; stage 2, head nodding; stage 3, rearing with unilateral tonic-clonic forelimb movements; stage 4, rearing with bilateral tonic-clonic forelimb movements; stage 5, rearing and falling with tonic-clonic forelimb movements. After induction of status epilepticus (SE: defined by the first stage 3–5 seizure that was not immediately followed by resumption of normal behavior), mice were placed in a new cage at room temperature for 2 h, then returned to the heated cage and seizure activity was reduced with injection of diazepam (10 mg/kg s.c.; Henry Schein). While sedated with diazepam, mice were injected with 5% dextrose-lactated Ringer’s solution (1 ml intraperitoneal; Henry Schein) and then returned to a clean cage the following morning, where they remained until sacrifice 5 weeks later. This timepoint was chosen to ensure that Pilo mice are in the chronic stages of epilepsy, which generally starts around 4 weeks post-Pilo when spontaneous recurrent seizures manifest (Botterill et al., 2019). Brain tissue was extracted and stored as described above for APP mice. All procedures were performed in accordance with Baylor College of Medicine and Nathan Kline Institute IACUC protocols.

### ΔFosB Chromatin Immunoprecipitation (ChIP)

To verify that hippocampal samples used for ΔFosB ChIP-seq came from mice with seizures and high ΔFosB expression, we first performed immunohistochemistry to detect ΔFosB in one hemibrain (right side) as described below. Following quantification and stratification of ΔFosB levels, mice were chosen for ChIP-seq based on robustness and uniformity of ΔFosB immunostaining. Mice for the APP/NTG analysis included 2 female APP mice with high levels of ΔFosB and 2 female NTG littermates (with typical baseline levels of ΔFosB), as previously described (Corbett et al., 2017; You et al., 2017, 2018). Mice for the Pilo/Sal analysis included 1 male/3 female Pilo mice with high levels of ΔFosB and 2 male/2 female Sal mice with baseline levels of ΔFosB as previously described (You et al., 2017). We isolated the entire hippocampal formation from each mouse by chilling each hemibrain (left side) on an ice block, and rolling out the entire hippocampal formation using a molt elevator (Roboz, RS-8830). To perform ChIP, after removing 2% of sheared chromatin samples to use as input, chromatin fragments bound to ΔFosB were isolated using a rabbit anti-ΔFosB antibody (Cell Signaling, D3S8R), with normal rabbit IgG (Millipore 12–370) used as a negative control. Western blot analysis of hippocampal lysates using D3S8R is shown in Supplementary Figure S1. All antibody concentrations were 2 μg/reaction, and samples were processed using the Magna ChIP A Kit (Millipore) for pulldown. To confirm ΔFosB binding at target genes in selected APP and Pilo mice prior to sample submission for sequencing, we performed qPCR to assess gene pulldown enrichment (vs. input) of ΔFosB at the *Calb1* promoter in our ChIP samples, and verified that binding of ΔFosB to *Calb1* in our ChIP samples was increased in the APP mice relative to NTG controls and in the Pilo mice relative to Sal controls, as we previously reported (You et al., 2017). Sequences of qPCR primers used to amplify the *Calb1* promoter were the following: 5’-TTCAAATACTCAACTGCCTCG-3’ (forward) and 5’-GGAGGCTTTCACTCCTGAATGT-3’ (reverse).

### ΔFosB Immunohistochemistry

Tissue preparation and immunohistochemistry were performed as previously described (Corbett et al., 2017; You et al., 2017). Hemibrains fixed with 4% formaldehyde were saturated for 48 h at 4°C with 30% sucrose in phosphate-buffered saline before being sectioned on a freezing, sliding microtome at a thickness of 30 μm, with ten coronal subseries collected throughout the rostral-caudal extent of the brain. Sections were stored at -20°C in cryoprotectant medium (30% glycerol, 30% ethylene glycol, 40% phosphate-buffered saline) until processing. For staining with 3,3-diaminobenzidine (DAB, Sigma-Aldrich), rabbit anti-ΔFosB (1:5000, Cell Signaling D3S8R) and biotinylated goat anti-rabbit secondary (1:200, Vector) were used, with further signal amplification using Avidin-Biotin Complex (Vector, ABC Elite). ΔFosB immunoreactivity was quantified by measuring mean pixel intensity in the dentate granule cell layer using ImageJ by an experimenter blinded with respect to genotype and/or treatment.

### ΔFosB ChIP-Sequencing, Read-Alignment, and Peak-Calling

Library preparation of ChIP-seq samples (NEBNext Kit, New England Biolabs) and single-read sequencing (50 bp read-depth) was performed by University of Pennsylvania Next-Generation Sequencing Core using Illumina hiSeq 2500 (NTG and APP samples) or 4000 (Sal and Pilo samples). Resulting FASTQ files were then uploaded to the next-generation sequencing analysis platform Basepair. Bowtie2 was used for alignment and quality control (all samples’ Q30 > 89%) using deduplicated reads, mapped onto the *Mus musculus* mm9 genome. Peak-calling was performed on aligned reads using MACS v2.1 (command: *callpeak –nomodel –extsize 200 –shift 0* with *p*-value < 1 × 10^–4^) and peak significance was determined via comparison of regional read enrichment between ΔFosB ChIP samples and no-antibody input samples (negative controls). Significant peaks were then annotated with name(s) of the nearest gene(s) and location data that denote binding to promoter (5-kb upstream of TSS), exon, intron, or intergenic regions. Individual lists of genes bound by significant ΔFosB peaks in animals of the same genotype or treatment-condition were pooled to generate all-inclusive lists of genes bound by ΔFosB in NTG, APP, Sal, or Pilo mice. R (v3.6.1; standard and *VennDiagram* packages) was used to generate lists and diagrams of genes that were uniquely bound in or overlap between lists from each group of mice.

### Gene Ontology (GO) Analysis

The Cytoscape platform (v3.7.1) app ClueGO (v2.5.4) was used to perform gene ontology (GO) analyses (Shannon et al., 2003; Bindea et al., 2009). Significant enrichment of Biological Process GO terms (as of 6/19/2019) was calculated for the specified gene lists using a two-sided hypergeometric test with a Benjamini-Hochberg correction (Benjamini and Hochberg, 1995). ClueGO was also used to generate functionally grouped networks of GO terms (nodes) enriched by ΔFosB target gene lists, with nodes connected by lines (edges) weighted to indicate the proportion of genes they share. Node size increases with greater significance of GO term enrichment. The functional clusters depicted for each network are those that are most representative and significantly enriched, simplified to remove redundancy. Additional ClueGO parameters were used to depict key aspects of each GO network, including kappa values that were set iteratively to generate clusters of comparable inclusivity/size, GO level minima set to exclude broad/vague GO terms from levels 1 to 2, GO level maxima set to include all significant GO terms regardless of detail level, and other parameters set to maximize detail and visual clarity. ClueGO network parameters changed from default are as follows: FDR < 0.05 (Supplementary Figures S2, S3) or FDR < 0.5 (Figure 3), GO level range = 3–20, minimum number of genes in term = 1, minimum percentage of genes in term = 0.1%, GO term fusion/grouping = on. Kappa varied between analyses: 0.59 (Supplementary Figure S2); 0.69 (Supplementary Figure S3); 0.67, % terms for Group merge = 70% (Figure 3).

### KEGG Pathway Analysis

The gene sets from Kyoto Encyclopedia of Genes and Genomes (KEGG) were examined to identify the pathways most significantly enriched by ΔFosB target genes. KEGG pathway enrichment was determined by hypergeometric test as implemented in the *clusterProfiler* package (v3.12.0) of R 3.6.1, with Benjamini-Hochberg adjusted *p*-value cutoff < 0.30 (Yu et al., 2012). A bubble chart of the enriched pathways was generated with the R package *ggplot2*. The size of each circle is proportional to its corresponding GeneRatio, which is calculated as the ratio of the number of target genes in the specific pathway relative to the total number of target genes in any KEGG pathways.

### RNA Extraction and qRT-PCR

RNA extraction was performed using adapted instructions from the Qiagen RNeasy Mini kit (74106). For these experiments, hippocampi were isolated from male and female 4-month old NTG or APP mice, or male and female 4-5-month old Sal or Pilo mice at 6–15 weeks post-injection. Samples included 18 NTG mice (6 females, 12 males) and 14 APP mice (6 females, 8 males), and 11 Sal mice (6 females, 5 males) and 11 Pilo mice (5 females, 6 males). Hippocampi were submerged in RLT/β-mercaptoethanol buffer, minced with small scissors, and homogenized by passing the lysate through a 21G needle 15 times. Samples were centrifuged and the supernatants were transferred to new tubes. RNA was then purified according to the kit instructions, and eluted with nuclease-free water. Final RNA concentration was determined using a NanoDrop One spectrophotometer. Reverse transcription was performed using the TaqMan Reverse Transcription Reagent kit (ABI, N8080234) in accordance with the manufacturer’s instructions, also adding 2.5 μM random hexamers and oligo d(T)_16_ per reaction (ABI, N8080127 and N8080128). The resulting cDNA was diluted in water and used for quantitative PCR, which was performed with an ABI StepOnePlus machine using SYBR Green (ABI, 4309155) as a fluorophore. Each sample was run in triplicate reactions. The primer sets listed below were used to amplify cDNA of the ΔFosB targets that are listed with respective sample size information and in forward (F) and reverse (R) format. Each primer pair was used at concentrations of 0.5 μM per reaction as follows: *Gapdh*: F, 5’-AATTCAACGGCACAGTCAAGGC-3’ and R, 5’-TACTCAGCACCGGCCTCACC-3’; *Ptpn5* [STEP61 isoforms]: F, 5’-GTCTCTCTGACTGTGAGC-3’ and R, 5’-TCT GAGTAGGTGAAGGAAGG; *Fat4*: F, 5’-ACACAACAG AGACATCGTC-3’ and R, 5’-CACCATAGAGCACTGAGG-3’; *Gmnc*: F, 5’-TAACTACAGTGCCACCAC-3’ and R, 5’-TTC CCTGACCTACATAGTCG-3’; *Cxcl12*: F, 5’-CGGTAAACC AGTCAGCCT-3’ and R, 5’-TCTGTTGTTGTTCTTCAGCC-3’.

### RNA-Sequencing

Approximately 300 ng of RNA extracted from the dentate gyrus of 4-month old APP mice with high ΔFosB expression and from wild-type NTG mice (4 per genotype, including 1 female, 3 male in each genotype) were submitted to the University of Pennsylvania Next-Generation Sequencing Core, where library preparation and Illumina hiSeq 2500 paired-read sequencing (100 bp read-depth) were performed. Data was uploaded to Basepair for analysis. In brief, reads were trimmed, aligned to mouse genome mm9, and counted using STAR and FeatureCounts. Differential expression analyses between genotypes were performed using DEseq, using sex as a secondary factor.

### Statistical Analyses

In general, statistical analyses were performed using SPSS-23 (IBM), Prism 8 (GraphPad), and R (v3.6.1). Sample sizes were determined based on accumulated empirical data and power analyses, and the numbers of samples used for each experiment were appropriate to detect significant differences. Unless otherwise stated, results are represented as sample means ± standard errors of the mean. Data are distributed normally, and differences between experimental groups were assessed by unpaired, two-tailed Student’s *t*-test when comparing means between two groups. No specific method of randomization was used, but animals were semi-randomly assigned to experimental groups based on birth order after balancing for age, sex, and genotype. Statistical significance of the overlap between two gene lists were evaluated with the hypergeometric test. Chi-square tests were also used as indicated in corresponding figure legends.

## Results

### Binding of ΔFosB to Target Genes Is Expanded and Diversified in APP Mice and Pilo Mice

**FIGURE 1:**
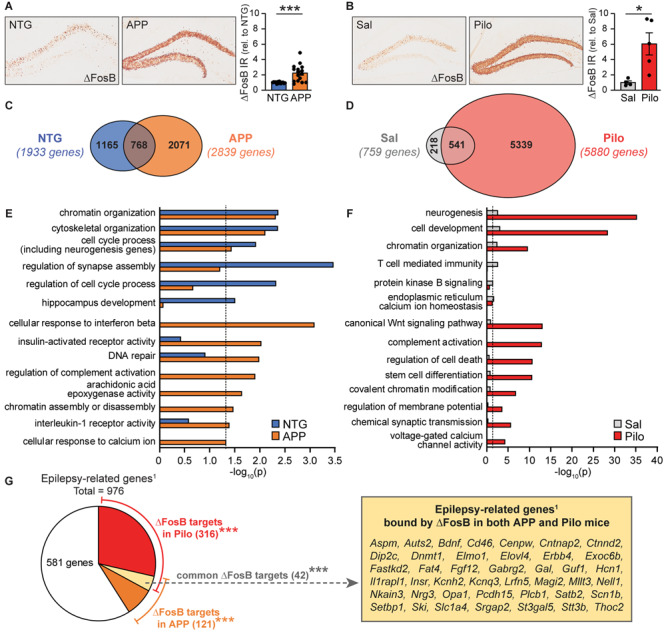
ΔFosB target gene binding and functional repertoires are expanded and diversified in APP and Pilo mice. **(A)** Example images (left panel) and quantification (right) of ΔFosB immunoreactivity in the dentate gyrus of the hippocampus in NTG and APP mice at 4 months of age (*n* = 15–16/genotype, ***p < 0.001 Student’s two-tailed unpaired *t*-test). **(B)** Example images (left panel) and quantification (right) of ΔFosB immunoreactivity in the dentate gyrus of the hippocampus in Sal and Pilo mice 5 weeks post-treatment (*n* = 4–5/group, *p < 0.05 Student’s two-tailed unpaired *t*-test). **(C)** Venn diagram representing the number of genes bound by ΔFosB in NTG and APP mice. **(D)** Venn diagram representing the number of genes bound by ΔFosB in Sal and Pilo mice. **(E,F)** Biological Process GO terms enriched by genes bound by ΔFosB in NTG and APP mice **(E)** and in Sal and Pilo mice **(F)**. Dotted lines indicate *p* = 0.05 (two-sided hypergeometric tests with Benjamini-Hochberg correction). **(G)** Schematic depicting the number of epilepsy-related genes published in Wang et al. (2017), that are also bound by ΔFosB in Pilo mice, APP mice, or both. ***p < 0.001 using hypergeometric test for overlap between known epilepsy-related genes^1^ with the list of ΔFosB target genes in Pilo mice, APP mice, or both. Call-out box lists the 42 genes bound by ΔFosB in both APP and Pilo mice that were present in an Epilepsy-related genes database (Wang et al., 2017).

We previously demonstrated that seizures increase expression of ΔFosB in the hippocampus of both APP mice and wild-type mice treated with pilocarpine (Corbett et al., 2017; You et al., 2017). Examples of ΔFosB expression and quantification of ΔFosB immunoreactivity are shown in Figures 1A,B. To assess the potential impacts of ΔFosB on gene regulation in different contexts with chronic recurrent seizures, we used chromatin immunoprecipitation and sequencing (ChIP-seq) to analyze ΔFosB-bound DNA in hippocampal lysates from 4-month old APP mice or non-transgenic (NTG) littermates. We identified significant ΔFosB binding peaks at 4,004 total genes in either NTG or APP mice, with 1,933 target genes bound in NTG mice vs. 2,839 target genes bound in APP mice (Figure 1C, full dataset is available in Supplementary Datasheet S1). Of the 4,004 total genes, 768 targets (19%) were bound in both NTG and APP mice. 1,165 genes (29%) were bound only in NTG mice compared to 2,071 genes (52%) bound only in APP mice.

We then performed a second ChIP-seq study using hippocampal lysates from wild-type mice treated with saline (Sal mice) or pilocarpine (Pilo mice; 5-weeks post-injection). We found significant ΔFosB binding peaks at 6,098 total genes in either Sal or Pilo mice, with 759 target genes bound in Sal mice vs. 5,880 target genes bound in Pilo mice (Figure 1D, full dataset is available in Supplementary Datasheet S2). Of the 6,098 total genes, 541 genes (9%) were bound in both Sal and Pilo mice, 218 genes (4%) were bound only in Sal mice, and 5,339 genes (87%) were bound only in Pilo mice. Overall, ΔFosB bound to more total genes in APP mice and in Pilo mice (vs. respective controls).

We used Gene Ontology (GO) analyses to characterize and compare the functions enriched by ΔFosB targets in NTG mice and APP mice (Figure 1E). Overarching GO Terms related to chromatin organization, development and cytoskeletal regulation, and cell cycle and neurogenesis were significantly enriched in both NTG and APP mice. We found that several terms were significantly enriched only in NTG mice, including regulation of synapse assembly, regulation of cell cycle process, and hippocampus development. Notably, many terms were significantly enriched only in APP mice, including those related to responses to immune signals, DNA repair, and calcium signaling.

GO analyses of functions enriched by ΔFosB targets in Sal and Pilo mice revealed overarching terms related to neurogenesis, cell development, and chromatin organization in both Sal and Pilo mice (Figure 1F). Several terms were significantly enriched only in Sal mice, including T-cell mediated immunity, protein kinase B signaling, and endoplasmic reticulum calcium homeostasis. Terms that were significantly enriched only in Pilo mice included those related to Wnt signaling, chromatin modifications, and excitability. We noted that both sets of GO Terms enriched in APP mice and in Pilo mice contain a high degree of representation from four key functional categories: (1) Excitability and Neurotransmission, (2) Neurogenesis, (3) Chromatin Remodeling, and (4) Cell Stress and Immunity (Supplementary Figures S2, S3).

We noted that these categories reflect neuronal functions that are typically altered in conditions with recurrent seizures; we therefore compared the lists of ΔFosB target genes in APP or Pilo mice to a published list of 976 genes that have putative or confirmed roles in epilepsy patients or mouse models used to study epilepsy (Figure 1G; Wang et al., 2017). We found that of the 2,839 genes bound by ΔFosB in APP mice, there was a significant overlap of 121 genes with epilepsy-related genes (*p* = 0.001). In addition, of the 5,880 genes bound by ΔFosB in Pilo mice, there was a significant overlap of 316 genes (*p* = 5.3 × 10^–22^). Intriguingly, of the 731 ΔFosB target genes bound in *both* APP and in Pilo mice, there was a significant overlap of 42 genes with the list of 976 seizure-related genes (*p* = 3.0 × 10^–4^). Together, these results indicate that the repertoires of ΔFosB target genes and functions are expanded in APP and Pilo mice relative to respective controls, and significant proportions of genes bound in APP mice and in Pilo mice fall under the category of epilepsy-related genes published in Wang et al. (2017).

### Seizure-Induced ΔFosB Was Bound to Most Targets in Ways Specific to Seizure Etiology, but ΔFosB Bound to Some Targets Regardless of Seizure Etiology

**FIGURE 2:**
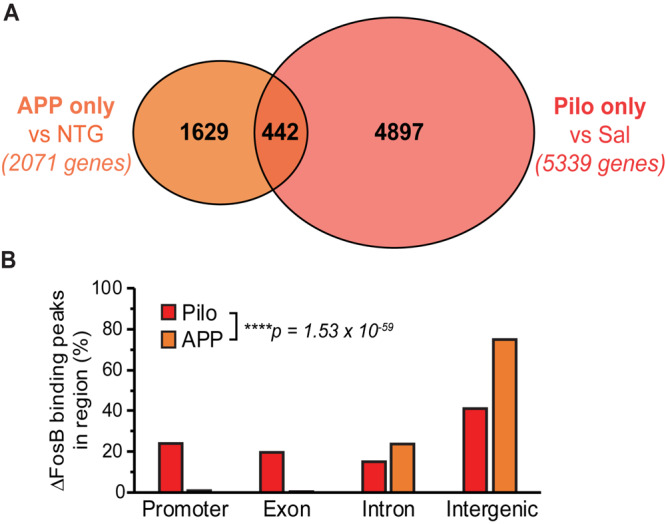
Binding of ΔFosB to target genes can depend on particular conditions within a disease context, but ΔFosB can also bind to some targets independently of seizure etiology. **(A)** Venn diagram of the number of genes bound by ΔFosB only in APP mice (relative to NTG mice), and those bound only in Pilo mice (relative to Sal mice). **(B)** Genomic distribution of ΔFosB binding peaks in the subset of 442 target genes common to APP and Pilo mice. ****p = 1.53 × 10^– 59^, Chi-square test.

The genes bound by ΔFosB in APP mice and in Pilo mice (but not in respective controls) provide information about which targets and pathways are regulated by ΔFosB in the context of recurrent seizures that occur in each condition. To gain perspective on which ΔFosB targets are bound regardless of seizure etiology, we compared the genes that were bound in APP but not NTG mice (2,071 genes) or bound in Pilo but not Sal mice (5,339 genes) and identified 442 overlapping ΔFosB targets bound in both APP and Pilo mice (but not in respective controls) (Figure 2A, full dataset is available in Supplementary Datasheet S3). In contrast, ΔFosB bound 1,629 genes uniquely in APP mice (but not in NTG or Pilo mice), and bound 4,897 genes uniquely in Pilo mice (but not in Sal or APP mice). This result indicates that seizure-induced ΔFosB binds targets in both seizure etiology-dependent and -independent manners. We found that, of the 442 genes bound by ΔFosB in both APP and Pilo mice, the distribution of the genomic regions to which ΔFosB was bound differed significantly between APP and Pilo mice (*p* = 1.53 × 10^–59^; Figure 2B). Pilo mice exhibited ΔFosB binding that was distributed similarly across promoter, exonic, intronic, and intergenic regions, but ΔFosB binding in APP mice was primarily focused at intronic and intergenic regions, similar to our previous ΔFosB ChIP-seq findings (You et al., 2018) and in general for transcription factor binding in other ChIP-seq studies of transcription factor binding, including that of some AP-1 factor complexes (Biddie et al., 2011; Slattery et al., 2014). The differing ΔFosB binding patterns in APP and Pilo mice suggest that ΔFosB may have multiple modes of binding that could be modulated by molecular and/or other factors that vary between disease contexts.

### Gene Ontology Analyses Reveal That ΔFosB Target Genes Bound in Both APP and Pilo Mice Relate to Pathways Implicated in AD and Other Diseases With Seizure Activity

**FIGURE 3:**
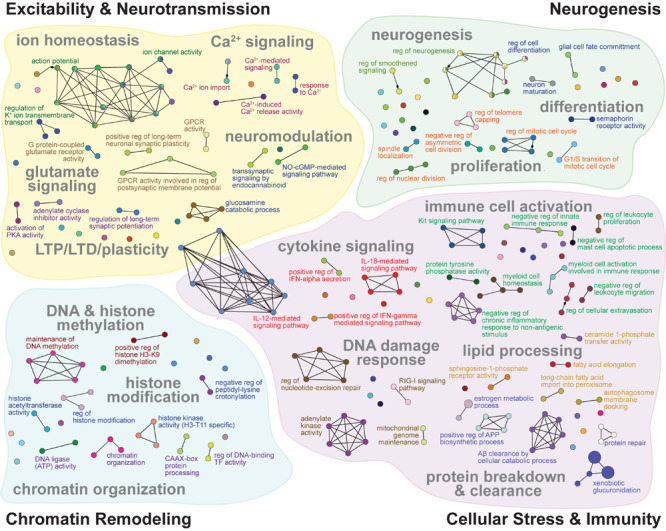
GO networks of representative terms related to excitability and neurotransmission, neurogenesis, chromatin remodeling, and cellular stress and immunity that are enriched by ΔFosB targets in both APP and Pilo mice. GO networks for the subset of 442 genes bound by ΔFosB in both APP and Pilo mice, grouped into Excitability and Neurotransmission (top left), Neurogenesis (top right), Chromatin Remodeling (bottom left), and Cellular Stress and Immunity (bottom right). GO terms (nodes) enriched with target genes are represented as circles, whose sizes increase with increasing statistical significance of the enrichment. Lines connect nodes with shared genes. Colored font indicates the leading term of the associated cluster. See Supplementary Table S1 for full listing of the GO Terms represented by each node pictured.

To assess the cellular functions that may be regulated by ΔFosB regardless of seizure etiology, we used ClueGO to construct a GO Network of the Biological Process GO Terms enriched by the 442 ΔFosB target genes that APP and Pilo mice share. We found that GO Terms related to AD and other diseases with seizure activity were indeed associated with the 442 genes bound in both APP and Pilo mice (Figure 3 and Table 1). The GO Network generated from these 442 genes (see Figure 3) was dominated by four categories: Excitability and Neurotransmission, Neurogenesis, Chromatin Remodeling, and Cellular Stress and Immunity. Notably, these four categories were the same as those highlighted in the GO networks of the total genes bound in either APP or Pilo mice (Supplementary Figures S2, S3). A full listing of all of the GO Terms represented in the GO Network in Figure 3 can be found in Supplementary Table S1.

**TABLE 1:** ΔFosB target genes bound in APP and Pilo mice are associated with GO Biological Processes that may influence AD and other diseases with recurrent seizures.

**Category**	**Select gene ontology**	**Annotated ΔFosB target genes**
	**(GO) Terms**	**(of 442 shared by APP and Pilo mice)**
Excitability and Neurotranmission	Action potential	*Dpp6, Dsc3, Fgf12, Hcn1, Kcnc2, Kcnh2, Met, Ryr2*
	Ion channel activity	*Dpp6, Fam155a, Fgf12, Gal, Grm5, Grm7, Gsg1l, Hcn1, Kcnc2, Kcnh2, Kcnq3, Klhl24, Ryr2, Slc1a4, Slc26a7, Trpc6, Vdac1*
	Glutamate receptor signaling pathway	*Grm5, Grm7, Gsg1l, Klhl24, Plcb1*
	Regulation of synaptic plasticity	*Adcy8, Bdnf, Ctnnd2, Cx3cr1, Grm5, Mme, Ptpn5/STEP61, Sorcs3, Stau1*
	Cellular lipid metabolic process	*Abcd3, Chd7, Cyp2c38, Elovl4, Hacd1, Mboat1, Pla2g4c, Plcb1, Prkag2, Sis, St8sia4*
	Hormone metabolic process	*Bmpr1b, Gal, Iqschfp, Psg18, Schip1, Srd5a2, Ugt1a10, Ugt1a7c*
Neurogenesis	Regulation of neurogenesis	*Asap1, Aspm, Bdnf, Chd7, Cx3cr1, Cxcl12, Dennd5a, Dlg5, Faim, Grm5, Hey2, Met, Mme, Nbn, Ncoa3, Ndrg4, Opa1, Plxna2, Plxna4, Ptpn5/STEP61, Rgs6, Rheb, Ski, Sox9, Srgap2, Syt17, Tnfrsf21, Trpc6, Vwc2*
	Cell cycle G2/M phase transition	*Cdc25a, Chek1, Hacd1, Nbn, Nek10, Plcb1*
	Regulation of stem cell proliferation	*Aspm, Bdnf, Cx3cr1, Ncoa3, Nfatc1, Pou3f4*
	Notch signaling pathway	*Cd46, Fat4, Galnt11, Hey2, Onecut1, Rps19, Sox9, Susd5, Xirp1*
	Smoothened signaling pathway	*B9d1, Dlg5, Fbxl17, Hhat, Rfx4, Tulp3*
	Cellular response to transforming growth factor beta stimulus	*Bmpr1b, Cx3cr1, Dnmt1, Ldlrad4, Onecut1, Ski, Sox6, Sox9*
	Melatonin receptor activity	*Gpr50*
Chromatin Remodeling	Chromatin organization	*Asxl2, Cdyl, Chd7, Chek1, Clock, Dmrtc2, Dnmt1, Epc1, H2al1n, Hmgb1, Hmgn3, Mbtd1, Mecom, Mysm1, Ncoa3, Satb1, Selenof, Setd6, Ski, Sox9, Trip12, Usp17le, Yeats2*
	Chromatin binding	*Gmnc, Hmgn3, Satb1, Nfatc1, Mef2a, Dnmt1, Onecut1, Ski, Sox9, Ncoa3*
	Histone H3-K9 modification	*Chek1, Dmrtc2, Dnmt1, Mecom*
	Histone acetylation	*Cdyl, Chek1, Clock, Epc1, Ncoa3, Yeats2*
	Monoubiquitinated histone H2A deubiquitination	*Mysm1*
	Maintenance of DNA methylation	*Dnmt1*
Cellular stress and immunity	Leukocyte activation	*Atp11c, Azi2, Camk4, Ccnd3, Cd46, Cd79a, Chd7, Crtc3, Cx3cr1, Cxcl12, Dlg5, Fer, Gab2, Gal, Hmgb1, Lat, Lrfn5, Met, Nbn, Ndrg1, Nfatc1, Nsfl1c, Onecut1, Satb1, Slc22a2, Slc39a10, Tnfrsf21, Tpd52*
	Myeloid cell differentiation	*Asxl2, Camk4, Gab2, Hmgb1, Inhba, Lbr, Nfatc1, Plcb1, Pou3f4, Rhd, Rps19, Slc25a38, Sox6*
	Oxidoreductase activity	*Cyp2c38, Lbr, Msrb2, Oxr1, Pah, Selenof, Sis, Srd5a2, Txnrd3*
	Amino acid transport	*Slc7a14*
	Autophagosome membrane docking	*Stx17*
	Amyloid-beta clearance by cellular catabolic process	*Mme* (neprilysin)

GO Terms involved in Excitability and Neurotransmission (Figure 3, top left) related to biological processes that regulate action potentials, intrinsic excitability, Na^+^, K^+^, Ca^2+^, and Cl^–^ channel activity and transmembrane transport, Ca^2+^ signaling, mechanisms of synaptic plasticity that include LTP and LTD, metabotropic glutamate receptor signaling, and inhibitory neuromodulation by nitric oxide, steroids, and endocannabinoids. Neurogenesis-related GO Term pathways (Figure 3, top right) included neuron and stem cell proliferation and differentiation, growth-factor secretion and receptor signaling, telomere capping, Notch signaling, and smoothened signaling. Chromatin Remodeling-related GO Terms (Figure 3, bottom left) contained pathways for classic processes that regulate chromatin architecture such as histone modification, DNA methylation, and alteration of chromosome topology. Cellular Stress and Immunity GO Terms (Figure 3, bottom right) represented diverse biological processes that can regulate apoptosis, calcineurin-NFAT signaling, Aβ degradation and clearance, microglial and leukocyte activation, complement signaling, interferon signaling, oxidoreductase activity, DNA and protein repair, inflammation, and protein synthesis.

### KEGG Analyses of ΔFosB Target Genes in Both APP and Pilo Mice Highlight Neurotransmission- and Neuroprotection-Related Pathways

**FIGURE 4:**
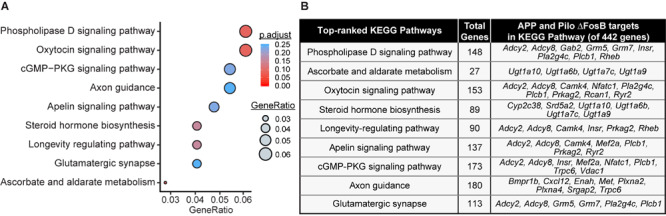
Pathway analysis of ΔFosB targets in APP and Pilo mice highlight neurotransmission and neuroprotection-related pathways. **(A)** KEGG pathway over-representation analysis on the subset of 442 target genes bound by ΔFosB in both APP and Pilo mice. Bubble chart shows enrichment of target genes in various signaling pathways (y-axis). Gene ratio (x-axis) is the ratio of the number of target genes enriched in the specific pathway relative to the number of target genes in any pathway. The gene ratio and *p*-value (calculated by hypergeometric test with Benjamini-Hochberg correction) of each pathway are indicated by the size and color of its corresponding circle, respectively. **(B)** List of target genes associated with each enriched KEGG pathway.

GO analyses provide broad coverage of cellular process annotations associated with the majority of protein-coding genes, but are limited in their ability to identify relationships between target genes in pathways. We therefore performed KEGG pathway analysis to identify pathways enriched by the 442 ΔFosB targets shared by APP and Pilo mice. Top-ranked KEGG pathways were primarily related to neurotransmission and intracellular signaling pathways (Figure 4). These KEGG pathways highlight a role for ΔFosB in control of neurotransmission and neuroprotection in diseases with seizures.

### Expression of Many ΔFosB Target Genes Is Altered in Hippocampal Tissue From APP and Pilo Mice Relative to Respective Controls

**FIGURE 5:**
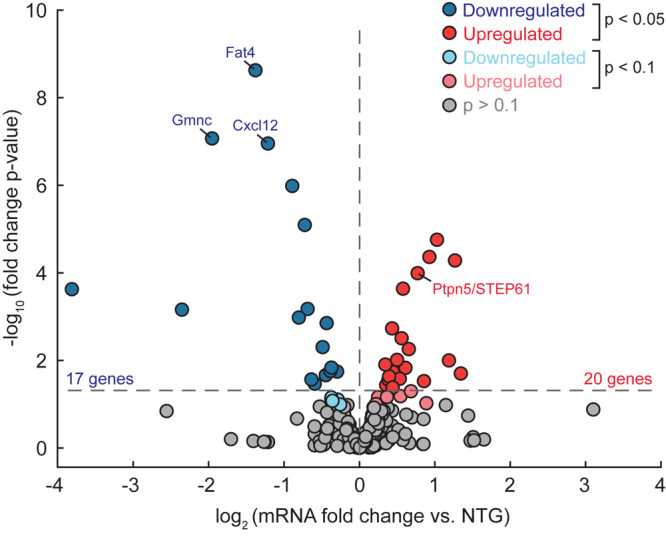
Some ΔFosB target genes bound in APP and Pilo mice are differentially expressed in the DG of APP mice (vs. NTG). RNA-seq was performed using dentate gyrus tissue from APP and NTG mice (*n* = 4/genotype), and differential gene expression was assessed using DESeq. Volcano plot illustrates gene expression data of the subset of 442 target genes bound by ΔFosB in both APP and Pilo mice. Horizontal dotted line indicates *p* < 0.05 threshold, while vertical dotted line separates upregulated genes from downregulated genes. Color of dot indicates significance level. Expression levels of labeled genes were independently assessed using RT-qPCR, as shown in Figure 6.

To begin to assess whether binding of ΔFosB to target genes is associated with corresponding changes in gene expression, we compared the list of 442 ΔFosB targets shared by APP and Pilo mice to an existing RNA-sequencing dataset that we had previously obtained from dentate gyrus (DG) tissue microdissected from an independent cohort of 4-month old APP and NTG mice. We assayed DG tissue in that case because ΔFosB accumulation is most robust in this region in mice with recurrent seizures (Corbett et al., 2017; You et al., 2017). In this cohort, APP mice were also confirmed to have high ΔFosB expression in DG via immunohistochemical staining of the contralateral hemibrain. We found that 20 genes were significantly upregulated and 17 genes were downregulated in the DG of APP mice relative to NTG mice (Figure 5 and Supplementary Datasheet S4).

**FIGURE 6:**
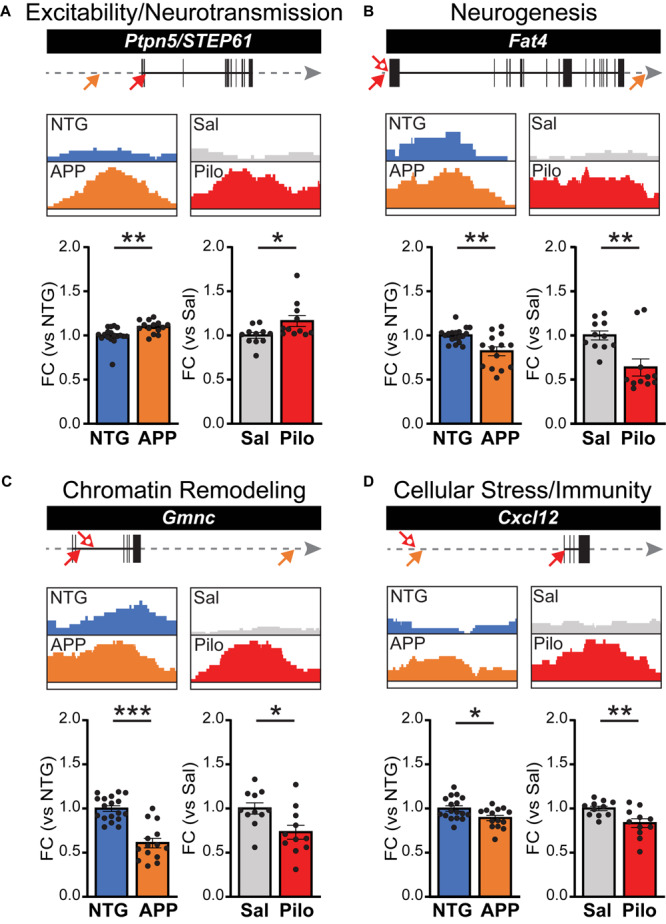
ΔFosB target genes bound in both APP mice and Pilo mice have altered mRNA expression levels. ΔFosB binding peaks and corresponding mRNA expression levels for example target genes from the subset of the 442 targets bound in both APP and Pilo mice. For each gene, locations of significant ΔFosB ChIP-seq binding peaks (*p* < 0.0001) in APP (orange) and Pilo (red) mice are marked on gene tracks by arrows (top; closed arrow, strongest peak; open arrow, additional peaks). Gray arrow indicates 135 kb stretch of genome. The strongest binding peaks in APP and Pilo mice, along with corresponding locations in their respective controls, are depicted in boxes (middle). Fold change (FC) of mRNA expression relative to respective controls (bottom) are shown for NTG and APP mice (left, *n* = 14–18/genotype) and Sal and Pilo mice (right, *n* = 11/group). **(A–D)** ΔFosB binding peaks and RT-qPCR mRNA expression levels for example genes related to **(A)** Excitability and Neurotransmission, **(B)** Neurogenesis, **(C)** Chromatin Remodeling, and **(D)** Cellular Stress and Immunity. *p < 0.05, **p < 0.01, ***p < 0.001, Student’s unpaired two-tailed *t*-tests.

To further confirm whether ΔFosB binding might affect transcription of its gene targets, we performed RT-qPCR using hippocampal lysates from additional independent cohorts of APP vs. NTG mice and Pilo vs. Sal mice. We assayed ΔFosB target genes from each of the four dominant categories described in Figure 3 and Table 1. For the Excitability and Neurotransmission category (Figure 6A), we examined *Ptpn5* (STriatal-Enriched protein tyrosine Phosphatase; primers detect STEP61 isoforms). We detected robust ΔFosB ChIP-seq binding peaks and increased mRNA expression this target gene in the hippocampus of APP mice relative to NTG mice. Significant binding peaks were also found in Pilo mice, as well as an increase in mRNA levels in Pilo relative to Sal mice. For the Neurogenesis category (Figure 6B), we found a significant binding peak in APP mice for *Fat4* (FAT atypical Cadherin 4) and a reduction in *Fat4* mRNA in APP relative to NTG mice, highlighting the bidirectional changes in gene expression that ΔFosB can induce (Robison and Nestler, 2011). Moreover, we found significant binding peaks as well as decreased *Fat4* mRNA in Pilo relative to Sal mice. For the Chromatin Remodeling category (Figure 6C), we assayed mRNA levels of *Gmnc* (Geminin Coiled-Coil Domain protein). We found robust ΔFosB ChIP-seq binding peaks in both APP and Pilo mice, as well as reduced *Gmnc* mRNA in APP mice and in Pilo mice relative to respective controls. In the Cellular Stress and Immunity category (Figure 6D) we examined *Cxcl12* (C-X-C motif chemokine ligand 12), which was significantly bound by ΔFosB in APP and Pilo mice. Expression of *Cxcl12* was decreased in APP mice relative to NTG controls, and in Pilo mice relative to Sal controls.

The APP mice examined thus far were 4 months old, at which age they have generally been having seizures for 6–8 weeks, given that we observe seizures beginning around 2–2.5 months old in this line of mice. To gain perspective on whether the regulation of ΔFosB target genes represents long-term modulation of expression, we also examined gene expression in APP/NTG mice at 2 months of age, when seizures have just begun. Notably, we found that at least for the genes listed above, similar magnitude and direction of change in expression were observed in APP mice at 2 months of age (Supplementary Figure S4). Taken together, these experiments suggest that ΔFosB binding to target genes exerts long-lasting control over their expression in APP and Pilo mice.

## Discussion

Neuronal dysfunction and memory deficits accompany conditions with recurrent seizures, and persist even when seizures are infrequent. Our results suggest that one mechanism by which seizures can cause such persistent impairments is via epigenetic regulation of gene expression, such as that induced by the actions of ΔFosB. Moreover, because the half-life of ΔFosB is unusually long (∼8 days *in vivo*), its impact on gene expression can be particularly long-lasting. Notably, we found that the repertoire of seizure-induced ΔFosB targets can depend on seizure etiology, but that there is also a subset of targets that are bound by ΔFosB independent of seizure etiology. The identification of this subset of genes may be valuable for identifying common pathways by which seizures affect neuronal function in varying diseases.

Our ChIP-seq studies identified unique and shared target genes of seizure-induced ΔFosB in a model of AD with recurrent seizures and high Aβ (APP mice) and in a model of TLE with recurrent seizures in the absence of AD-related factors (Pilo mice). This design generated three major sets of ΔFosB target genes that each addressed a different question: (1) which genes does ΔFosB bind in an AD mouse model (i.e., conditions with recurrent seizures and high Aβ), (2) which genes does ΔFosB bind in a TLE mouse model (i.e., conditions with recurrent seizures in the absence of AD-related factors), and (3) which genes does ΔFosB bind across different conditions with recurrent seizures? We identified ΔFosB target genes that are uniquely bound in either APP or Pilo mice that represent seizure etiology-dependent targets. Target selection may be influenced by the duration of seizures in each context and/or by context-specific factors such as AP-1 binding partner abundance, redox state, enhancer selection, recent firing activity, and chromatin architecture (Hai and Curran, 1991; Jorissen et al., 2007; Malik et al., 2014; Su et al., 2017; Vierbuchen et al., 2017; Yin et al., 2017; Jaeger et al., 2018; Fernandez-Albert et al., 2019; Nord and West, 2019). Indeed, activity-dependent alterations in chromatin accessibility may contribute to the differential patterns of ΔFosB binding mode (the distribution of ΔFosB binding sites in the gene) observed between APP and Pilo mice (Figure 2B), since Pilo mice have markedly more frequent and severe seizures than do APP mice. Conversely, ΔFosB target genes bound in both APP and Pilo mice (the subset of 442 genes) represent targets that are seizure etiology-independent, which are bound regardless of the context. Further investigation is required to identify which factors are most influential in shaping ΔFosB binding patterns in different contexts.

We found that many ΔFosB target genes are uniquely bound in APP mice or in Pilo mice, but each set of unique target genes (and the target genes that APP and Pilo mice share) contained some genes that are implicated in neurodegenerative diseases and/or epileptogenesis, and many genes that represent similar functional categories relevant to conditions with seizure activity (see Table 1). The KEGG and GO results highlighted Excitability and Neurotransmission, Neurogenesis, Chromatin Remodeling, and Cellular Stress and Immunity. Regardless of seizure etiology or disease context, ΔFosB appears to regulate consistent sets of *functions*, but enacts them through different *pathways.*

Our ChIP-seq studies used a relatively small number of animals made up of more females than males, which should be taken into account when considering these datasets. However, it is important to note that many of the genes were confirmed to be regulated at the expression level when assessed in independent cohorts with much larger numbers of animals that were made up of similar numbers of males and females (Figure 6 and Supplementary Figure S4). Therefore, while the possibility of sex-bias cannot be excluded, it does not appear to affect all of the results and interpretations in our study.

ΔFosB bound many genes in APP and Pilo mice that regulate key pathways that may influence pathophysiology in AD and other diseases with seizure activity. Interestingly, we noted that for some of the ΔFosB target genes that we examined with RT-qPCR and in our RNA-seq study, the direction of change in expression suggested that the actions of ΔFosB may be neuroprotective. For example, STEP61 was increased in APP mice and in Pilo mice relative to respective control mice (Figure 6A). We previously found that the STEP61 protein was also increased in the hippocampus in APP mice and kainate-treated wild-type mice with seizures, which corresponded with a neuroprotective reduction in phosphorylation and activity of Fyn kinase, and reduced phosphorylation of the NR2B receptor (Chin et al., 2005; Palop et al., 2007). In addition, ΔFosB may coordinate and balance target gene expression to achieve consistent combinatorial control of neuronal function across multiple molecular contexts. Such actions are similar to the manner in which the activity-induced transcription factor Npas4 maintains circuit excitation-inhibition balance via distinct programs of epigenetic regulation in different cell types and neuronal activity patterns (Spiegel et al., 2014; Brigidi et al., 2019). This diversity of tactics by which ΔFosB may regulate neuronal function underscores the therapeutic importance of determining which of these pathways may be most amenable to regulation in distinct conditions with seizures.

Surprisingly, even though RNA-seq of DG tissue and RT-qPCR of whole-hippocampus revealed changes in disease-critical ΔFosB target genes in APP and Pilo mice, many of the 442 genes bound by ΔFosB in APP and Pilo mice did not show significant changes in the RNA-seq study. We found that 8.4% of the 442 genes exhibited significant changes (*p* < 0.05) in gene expression, and 10.6% of genes at the *p* < 0.1 level. One obvious reason may be because the region sampled was different. Another factor may be the relatively small sample sizes in the RNA-seq study that included 4 animals per genotype. However, another possibility may be that only a subset of ΔFosB target genes are capable of exhibiting alterations in expression after seizures induce high levels of ΔFosB. A precedent for such dynamic, selective regulation of a subset of target genes has been observed with targets of REST/NRSF that is induced after seizures, wherein only ∼10% of potential NRSF target genes were repressed in a pharmacological epilepsy model (McClelland et al., 2014). Notably, in that study, genes that were selectively repressed by REST/NRSF had mid-range binding (e.g., percent input in ChIP studies) to REST/NRSF, whereas those that did not change in expression had either low- or high-range binding. Such mid-range binding frequencies were hypothesized to render those genes most sensitive to fluctuations in REST/NRSF levels after seizures. We have not investigated factors that impact binding frequency or whether the binding frequency of ΔFosB to its potential target genes shapes the likelihood that gene will exhibit altered expression, but these influences each remain an intriguing possibility.

An additional reason why many genes in the 442-gene subset of ΔFosB target genes might not have exhibited altered expression in APP mice in the RNA-seq study might relate to the possibility that ΔFosB (as a part of the AP-1 transcription factor complex) may also act to *maintain* baseline-level expression of its target genes in conditions with seizures as a way of protecting neuronal homeostasis by stabilizing gene expression. AP-1 has recently been discovered as a “pioneer” transcription factor complex that is required to permit stable target gene expression across different molecular contexts by opening and maintaining chromatin accessibility at target genes and enhancers, without necessarily altering gene expression (Biddie et al., 2011; Vierbuchen et al., 2017; Bejjani et al., 2019; Iwafuchi-Doi, 2019). Because other AP-1 factors like Fos and FosB are induced by neuronal and seizure activity and exhibit increased binding in ways required to maintain open chromatin at gene and enhancer regions after neuronal activity (Malik et al., 2014; Su et al., 2017; Fernandez-Albert et al., 2019), it is possible that ΔFosB can also participate in these activity-induced pioneer processes via dimerization with AP-1 factors. Due to ΔFosB’s long half-life, such a mechanism may contribute to the mechanisms by which AP-1 can maintain open chromatin states for at least 48 h (i.e., past the time-points at which AP-1 expression has subsided) (Su et al., 2017; Fernandez-Albert et al., 2019). If so, ΔFosB may serve along with other AP-1 factors as a long-lasting pioneer transcription factor induced by seizures that can maintain open chromatin and stabilize target gene expression in periods of recurrent seizures. In conclusion, our results are consistent with the possibility that ΔFosB binds to target genes that can either remain consistent or vary between diseases with different seizure etiologies, but which regulate consistent domains of neuronal function relevant to epileptogenesis and Aβ-induced pathophysiology.

## Data Availability Statement

The datasets generated for this study can be found in the ChIP-seq data: ArrayExpress accession E-MTAB-8954 and RNA-seq data: ArrayExpress accession E-MTAB-8963.

## Ethics Statement

The animal study was reviewed and approved by Institutional Animal Care and Use Committees of Baylor College of Medicine and the Nathan Kline Institute.

## Author Contributions

GS, C-HF, YL, and JC designed the research. GS, C-HF, CS, and YZ performed experiments. GS, C-HF, CS, YZ, and YL performed the data analyses. GS, C-HF, and JB generated research reagents. HS, YL, and JC supervised the project. GS, C-HF, HS, YL, and JC wrote the manuscript. All authors contributed to manuscript revision, read, and approved the submitted manuscript.

## Conflict of Interest

The authors declare that the research was conducted in the absence of any commercial or financial relationships that could be construed as a potential conflict of interest.
