# Supplementary material for: Genes Bound by ΔFosB in Different Conditions With Recurrent Seizures Regulate Similar Neuronal Functions
Source: Front Neurosci. 2020 May 28;14:472. doi: 10.3389/fnins.2020.00472 (PMC7268090; doi:10.3389/fnins.2020.00472)
Supplement: Supplementary file 5 [file Image_1.PDF]

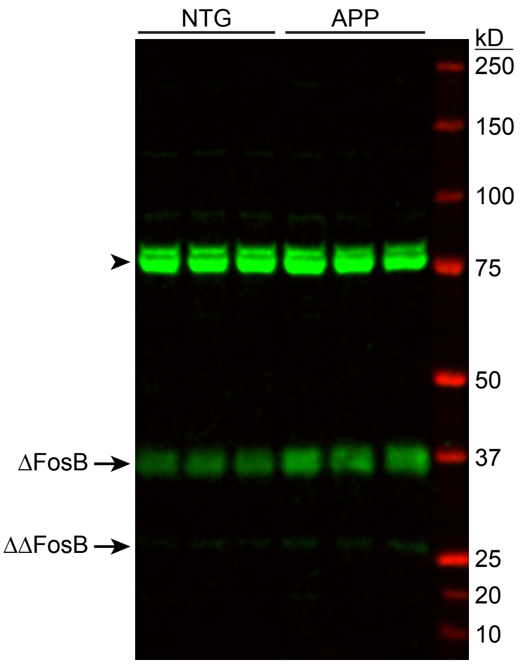

Supplemental Figure S1. Anti-ΔFosB antibody D3S8R (Cell Signaling, 1:3000) was used to probe a Western blot of hippocampal lysates (40 micro-grams protein/lane) from NTG and APP mice. Arrows denote bands for Δ FosB and possible Δ2ΔFosB, levels of which appear very low or negligible in hippocampus as we previously found (Corbett et al., 2017). Arrowhead denotes possible nonspecific band around 80-85kD. Note that the intensity of the 80-85 kD band does not differ between genotypes. The ΔFosB band at 37 kD is increased in intensity in APP mice relative to NTG littermate controls, as expected, suggesting that genotype or treatment specific differences in target genes pulled down by this antibody are likely due to ΔFosB binding.
