# Supplementary material for: Genes Bound by ΔFosB in Different Conditions With Recurrent Seizures Regulate Similar Neuronal Functions
Source: Front Neurosci. 2020 May 28;14:472. doi: 10.3389/fnins.2020.00472 (PMC7268090; doi:10.3389/fnins.2020.00472)
Supplement: Supplementary file 6 [file Image_2.PDF]

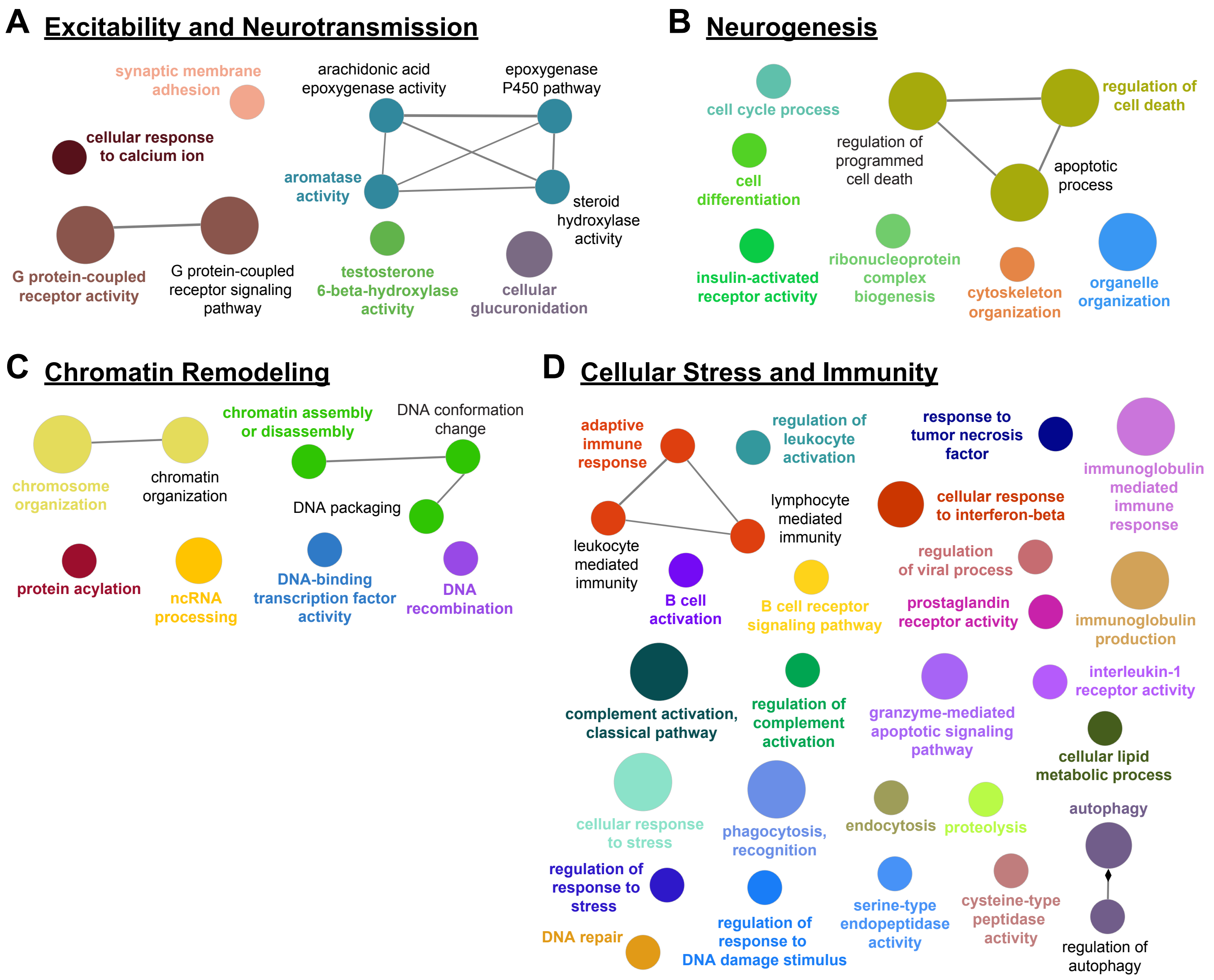

Supplemental Figure S2. Simplified GO networks of representative terms related to (**A**) Excitability and Neurotransmission, (**B**) Neurogenesis, (**C**) Chromatin Remodeling, and (**D**) Cellular Stress and Immunity that are significantly ($p < 0.05$) enriched by the 2,839 ΔFosB targets in APP mice.
