# Supplementary material for: Genes Bound by ΔFosB in Different Conditions With Recurrent Seizures Regulate Similar Neuronal Functions
Source: Front Neurosci. 2020 May 28;14:472. doi: 10.3389/fnins.2020.00472 (PMC7268090; doi:10.3389/fnins.2020.00472)
Supplement: Supplementary file 8 [file Image_4.PDF]

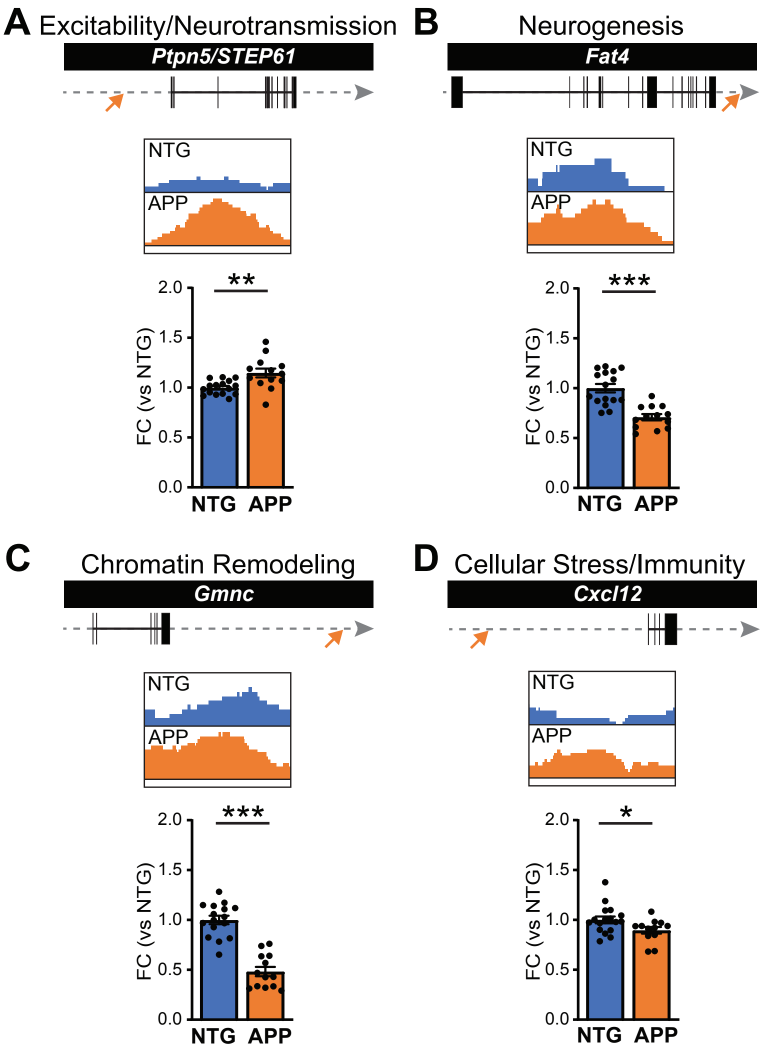

Supplemental Figure S4. ∆FosB target genes bound in hippocampus of APP mice have altered mRNA expression levels in mice at 2 months of age. ∆FosB binding peaks for example target genes shown in Figure 6 of the main paper, and mRNA expression levels in NTG and APP mice at 2 months of age. For each gene, locations of significant ∆FosB ChIP-seq binding peaks ($p < 0.0001$) in APP mice (orange) are marked on gene tracks by arrows. Gray arrow indicates 135 kb stretch of genome. The strongest binding peaks in APP mice, along with corresponding locations in NTG mice, are depicted in boxes (middle). Fold changes (FC) of mRNA expression in APP mice relative to NTG mice (bottom) are shown. (A-D) ∆FosB binding peaks and RT-qPCR mRNA expression levels for example genes related to (A) Excitability/Neurotransmission, (B) Neurogenesis, (C) Chromatin Remodeling, and (D) Cellular Stress/Immunity. *$p < 0.05$, *$p < 0.01$, ***$p < 0.001$, Student's unpaired two-tailed t-tests. n = 20 (NTG; 10 female, 10 male) and n = 15 (APP; 7 female, 8 male).
