# Supplementary material for: Genes Bound by ΔFosB in Different Conditions With Recurrent Seizures Regulate Similar Neuronal Functions
Source: Front Neurosci. 2020 May 28;14:472. doi: 10.3389/fnins.2020.00472 (PMC7268090; doi:10.3389/fnins.2020.00472)
Supplement: Supplementary file 9 [file Table_1.PDF]

Table S1. Full listing of GO Terms represented in network nodes in Figure 3

Excitability and Neurotransmission

Action potential, regulation of action potential, positive regulation of ion transmembrane transport, positive regulation of ion transport, regulation of cation transmembrane transport

Activation of PKA activity, glucose-mediated signaling pathway

Adenylate cyclase inhibitor activity, group III metabotropic glutamate receptor activity

Ca^{2+} ion import, regulation of Ca^{2+} ion import

Ca^{2+}-induced Ca^{2+} release activity, positive regulation of ATPase-coupled Ca^{2+} transmembrane transporter activity

Ca^{2+}-mediated signaling, second-messenger-mediated signaling

G protein-coupled glutamate receptor activity, G protein-coupled glutamate receptor signaling pathway

Glucosamine catabolic process, N-acetylneuraminate catabolic process, N-acetylglucosamine catabolic process, UDP-N-acetylglucosamine biosynthetic process

GPCR activity involved in regulation of postsynaptic membrane potential, neurotransmitter receptor activity involved in regulation of postsynaptic cytosolic Ca^{2+} ion concentration, regulation of intracellular Ca^{2+} activated Cl^- channel activity

GPCR activity, GPCR signaling pathway

Ion channel activity, cation transmembrane transport, regulation of ion transmembrane transport

NO-cGMP-mediated signaling pathway, voltage-gated ion channel activity involved in regulation of presynaptic membrane potential

Positive regulation of long-term neuronal synaptic plasticity, trans-synaptic signaling, modifying synaptic transmission

Regulation of K^+ ion transmembrane transport, K^+ channel activity, regulation of K^+ ion transmembrane transporter activity

Regulation of long-term synaptic potentiation, regulation of synaptic plasticity

Response to Ca^{2+}, cellular response to Ca^{2+} ion

Trans-synaptic signaling by endocannabinoid, postsynaptic modulation of chemical synaptic transmission

Singlet GO Terms: anion channel activity, Ca^{2+}-mediated signaling using intracellular Ca^{2+} source, calmodulin-dependent protein kinase activity, glutamate receptor signaling pathway, membrane hyperpolarization, modification of synaptic structure, negative regulation of synaptic transmission, postsynapse to nucleus signaling pathway, regulation of long-term synaptic depression, regulation of receptor internalization, regulation of synaptic plasticity by receptor localization to synapse, retrograde trans-synaptic signaling by neuropeptide modulating synaptic transmission

Neurogenesis

G1/S transition of mitotic cell cycle, positive regulation of mitotic cell cycle phase transition

Glial cell fate commitment, positive regulation of mesenchymal stem cell differentiation

Negative regulation of asymmetric cell division, maintenance of centrosome location

Neuron maturation, cell dedifferentiation

Regulation of cell differentiation, positive regulation of cell development

Regulation of mitotic cell cycle, mitotic cell cycle, regulation of cell cycle, mitotic cell cycle process

Regulation of neurogenesis, neurogenesis, neuron differentiation, cell development

Regulation of nuclear division, positive regulation of nuclear division, positive regulation of glycoprotein biosynthetic process

Regulation of smoothened signaling pathway, smoothened signaling pathway

Regulation of telomere capping, positive regulation of telomere maintenance, positive regulation of telomere maintenance via telomerase

Semaphorin receptor activity, semaphorin-plexin signaling pathway involved in axon guidance

Spindle localization, establishment of spindle orientation

Singlet GO Terms: dorsal/ventral pattern formation, forebrain generation of neurons, GABAergic neuron differentiation, growth factor activity, multi-ciliated epithelial cell differentiation, negative regulation of BMP signaling pathway, negative regulation of maintenance of mitotic sister chromatid cohesion (telomeric), negative regulation of stem cell proliferation, Notch signaling pathway, oligodendrocyte cell fate specification, positive regulation of stem cell proliferation, positive regulation of Wnt signaling pathway (planar cell polarity pathway), programmed cell death involved in cell development, regulation of stem cell differentiation, regulation of stem cell proliferation, stem cell differentiation, traversing start control point of mitotic cell cycle

Chromatin Remodeling

CAAX-box protein processing, positive regulation of MDA-5 signaling pathway

Chromatin organization, peptidyl-lysine modification, histone modification

DNA ligase (ATP) activity, Okazaki fragment processing involved in mitotic DNA replication

Histone acetyltransferase activity, histone acetylation

Histone kinase activity (H3-T11 specific), DNA damage-induced protein phosphorylation, regulation of transcription from RNA polymerase II promoter in response to UV-induced DNA damage

Maintenance of DNA methylation, DNA hypermethylation, positive regulation of methylation-dependent chromatin silencing, DNA methylation on cytosine within a CG sequence, DNA (cytosine-5-)-methyltransferase activity acting on CpG substrates

Negative regulation of peptidyl-lysine crotonylation, random inactivation of X chromosome

Positive regulation of histone H3-K9 dimethylation, positive regulation of histone H3-K9 trimethylation

Regulation of DNA-binding transcription factor activity, positive regulation of DNA-binding transcription factor activity

Regulation of histone modification, negative regulation of histone modification, negative regulation of chromosome organization

Singlet GO Terms: positive regulation of histone H3-K27 trimethylation, DNA ligation, DNA-binding transcription activator activity (RNA polymerase II-specific), DNA-binding transcription factor activity, histone H3-K9 modification, monoubiquitinated histone H2A deubiquitination, negative regulation of histone deacetylation, negative regulation of histone H2A K63-linked ubiquitination, nucleus organization, positive regulation of histone methylation

Cellular Stress and Immunity

Adenylate kinase activity, nucleoside triphosphate adenylate kinase activity, ITP metabolic process, AMP phosphorylation, ADP biosynthetic process, dATP metabolic process

Autophagosome membrane docking, endoplasmic reticulum-Golgi intermediate compartment organization

Aβ clearance by cellular catabolic process, oligopeptidase activity, neuropeptide processing, cellular response to UV-A, cellular response to UV-B, negative regulation of endothelial cell-matrix adhesion via fibronectin

Ceramide 1-phosphate transfer activity, intermembrane lipid transfer

Estrogen metabolic process, hormone metabolic process

Fatty acid elongation, very long-chain fatty acid metabolic process

IL-12-mediated signaling pathway, IL-15-medated signaling pathway, cellular response to ionomycin, regulation of transsynaptic signaling by endocannabinoid, positive regulation of sodium:proton antiporter activity, cellular response to vasopressin, cellular response to glyceraldehyde, cellular response to fluoride, positive regulation of CD24 biosynthetic process

IL-18-mediated signaling pathway, paracrine signaling, negative regulation of phosphatidylinositol biosynthetic process, superoxide-generating NADPH oxidase activator activity

Kit signaling pathway, F_c-epsilon receptor signaling pathway, diapedesis, insulin receptor signaling pathway via phosphatidylinositol 3-kinase

Long-chain fatty acid import into peroxisome, very long-chain fatty acid catabolic process

Mitochondrial genome maintenance, mitochondrion distribution

Myeloid cell activation involved in immune response, mast cell activation, leukocyte degranulation

Myeloid cell homeostasis, myeloid cell differentiation, homeostasis of number of cells, erythrocyte differentiation

Negative regulation of chronic inflammatory response to non-antigenic stimulus, positive regulation of I-kB phosphorylation, negative regulation of hippocampal neuron apoptotic process, central nervous system maturation, multiple spine synapse organization (single dendrite)

Negative regulation of innate immune response, negative regulation of immune response, lymphocyte-mediated immunity

Negative regulation of leukocyte migration, regulation of mononuclear cell migration

Negative regulation of mast cell apoptotic process, positive regulation of mast cell proliferation

Positive regulation of APP biosynthetic process, thrombin-activated receptor activity, positive regulation of hydrogen peroxide-mediated programmed cell death, negative regulation of hydrogen peroxide-mediated programmed cell death

Positive regulation of interferon-alpha secretion, interferon-alpha production

Positive regulation of interferon-gamma-mediated signaling pathway, positive regulation of MHC class I biosynthetic process

Protein repair, peptide-methionine (R)-S-oxide reductase activity, L-methionine-(R)-S-oxide reductase activity

Protein tyrosine phosphatase activity, phosphoprotein phosphatase activity

Regulation of cellular extravasation, cellular extravasation, leukocyte rolling or tethering

Regulation of leukocyte proliferation, negative regulation of lymphocyte proliferation

Regulation of nucleotide excision repair, Ca^{2+}-dependent protein kinase regulator activity, positive regulation of toll-like receptor 9 signaling pathway, regulation of T cell-mediated response to tumor cell, plasmacytoid dendritic cell activation

RIG-I signaling pathway, cellular response to virus

Sphingosine-1-phosphate receptor activity, positive regulation of establishment of endothelial barrier

Xenobiotic glucuronidation, flavonoid glucuronidation, xenobiotic metabolic process, cellular response to xenobiotic stimulus

Singlet GO Terms: 3’-5’-exoribonuclease activity, activation of protein kinase B activity, calcineurin-NFAT signaling cascade, C-C chemokine receptor activity, chemokine (C-X-C motif) ligand 12 signaling pathway, endopeptidase activity, endosome fission, G2 DNA damage checkpoint, macrophage activation, myeloid leukocyte activation, neutrophil homeostasis, oligodendrocyte apoptotic process, oxidoreductase activity, positive regulation of IL-1β secretion, protein quality control for misfolded or incompletely synthesized proteins, regulation of glucocorticoid receptor signaling pathway, regulation of IL-10 production, regulation of protein stability, regulation of TGF-β production, TGF-β receptor activity
